# Childhood Obesity Surveillance Initiative (COSI) in Poland: Implementation of Two Rounds of the Study in the Context of International Methodological Assumptions

**DOI:** 10.34763/jmotherandchild.2020241.1936.000001

**Published:** 2020-07-29

**Authors:** Anna Fijałkowska, Anna Dzielska, Joanna Mazur, Magdalena Korzycka, Joao Breda, Anna Oblacińska

**Affiliations:** 1 Department of Cardiology, Institute of Mother and Child, Warsaw, Poland; 2 Department of Child and Adolescent Health, Institute of Mother and Child, Warsaw, Poland; 3 Department of Humanization in Medicine and Sexology, Collegium Medicum, University of Zielona Gora, Zielona Gora, Poland; 4 WHO Regional Office for Europe, Copenhagen, Denmark

**Keywords:** body weight, children, overweight, obesity, blood pressure, epidemiology

## Abstract

Nowadays, childhood obesity is one of the key health problems in European countries. This article presents a study that is part of the World Health Organization (WHO) Childhood Obesity Surveillance Initiative (COSI) implemented in the WHO European Region since 2007. The main goal of the study is to monitor obesity in early school-aged children. The methodology of the study, the thematic scope of research tools, the organisational principles and the development of research carried out in Poland in the context of existing international assumptions are presented. In Poland, two rounds of the study were financed by the National Health Program, in cooperation with the WHO Office in Poland. The first study was carried out from November to December 2016 on a group of 3,408 children aged 8 years from 135 schools and 2,298 parents, in 9 voivodeships in Poland. The second round was carried out in the last quarter of 2018 in 12 voivodeships. A group of 2691 pupils aged 8 years from the 2nd grade of 140 primary schools in Poland and 2450 parents were examined. Data on body mass index distribution and lifestyle-related behaviours of children and their families were collected. Poland is the first country where blood pressure was measured in all participants of the COSI study. Considering the growing obesity epidemic, reliable monitoring of overweight and obesity in early childhood and the study of determinants of this phenomenon should be a priority for public health. The results obtained from this type of research are a reference point for the design and implementation of accurate prevention initiatives in this age group.

## Streszczenie

Współcześnie otyłość stanowi jeden z kluczowych problemów zdrowotnych w krajach rozwiniętych. W pracy zaprezentowano badanie będące częścią międzynarodowego projektu Światowej Organizacji Zdrowia, Childhood Obesity Surveillance Initiative, realizowanego w krajach Regionu Europejskiego od 2007 roku. Głównym celem badań jest monitorowanie otyłości u dzieci w wieku wczesnoszkolnym. Przedstawiono założenia metodologiczne, zakres tematyczny narzędzi badawczych, zasady organizacji i przebieg badań realizowanych w Polsce w kontekście obowiązujących założeń międzynarodowych. W Polsce przeprowadzono dwie rundy badań finansowane przez Narodowy Program Zdrowia, we współpracy z Polskim Biurem WHO. Pierwsze badanie realizowano na terenie dziewięciu województw w okresie od listopada do grudnia 2016 r. w grupie 3408 dzieci w wieku 8 lat ze 135 szkół oraz 2298 rodziców. Drugą rundę badań przeprowadzono w ostatnim kwartale 2018 r. w zwiększonej liczbie 12 województw. Zbadano 2691 uczniów w wieku 8 lat ze 140 szkół podstawowych w Polsce oraz 2450 rodziców. Zebrano, między innymi, dane umożliwiające ocenę rozkładu wskaźnika masy ciała oraz stylu życia dzieci i ich rodzin. Jak dotychczas, tylko w Polsce wykonano również pomiary ciśnienia tętniczego krwi u wszystkich uczestników badania. W obliczu narastającej epidemii otyłości, rzetelne monitorowanie nadwagi i otyłości w okresie wczesnego dzieciństwa oraz badanie uwarunkowań tego zjawiska, powinno stanowić priorytet zdrowia publicznego. Wyniki uzyskane z tego rodzaju badań stanowią punkt odniesienia do projektowania i wdrażania trafnych działań profilaktycznych w tej grupie wieku, sprzyjając ograniczeniu negatywnych konsekwencji zdrowotnych i ekonomicznych otyłości w przyszłości.

## Introduction

Childhood obesity is associated with significant health problems, and it is an important early risk factor for much adult morbidity and mortality (1). The consequences of obesity include diseases and states affecting almost all systems and organs. Among the more common consequences of childhood obesity in future life are type 2 diabetes, elevated blood pressure, asthma, cardiovascular diseases, depression and problems with the skeletal system (2). The percentage of children who are obese or overweight at the beginning of their school education is a common indicator of childhood health (3). Overweight and obesity in children are also associated with a higher risk of psychosocial and emotional problems, antisocial behaviour, disturbances in attention and related school problems, as well as problems in relationships with peers (4,5).

Obesity is difficult to treat at all ages, and it is a long-lasting condition. Health problems that begin in childhood intensify with age. The rise in child obesity will almost certainly lead to a rise in adult obesity rates. Moreover, the occurrence of obesity in childhood predicts the risk of developing obesity during adolescence and, later, in adulthood (6–7). The effects of obesity in the early stages of development are also significant from the economical point of view. According to Hamilton et al. (8), the longer a person has been obese, greater are the excess morbidity and related costs caused by obesity in adulthood, especially if obesity began in adolescence or childhood.

In Poland, studies on the prevalence of overweight and obesity among children and adolescents have been conducted for years; however, the majority included children from selected cities or regions, or from different age groups (9, 10, 11, 12). Only a few studies analysed nationwide data of a representative nature. Research conducted in 2007–2009 among children aged 7–12 years under the OLAF study, for instance, aimed to create national standards against which to assess the standards of growth of children and adolescents (13). Another study was conducted in 2001 under the European Childhood Obesity Group (ECOG) project, among Polish children aged 7–9 years (14), and another was conducted in 2005 among adolescents aged 13–15 years from five regions of Poland, using anthropometric measurements (12). Finally, the Health Behaviours in School-aged Children (HBSC) study, which began in the 1990s, is a reliable source of data on Polish adolescent health. The long research tradition and unified methodology allow the trends in overweight and obesity among European youth aged 11–15 years to be followed, among other health indicators (15).

The studies cited above are a valuable source of information on the prevalence of overweight and obesity in children, but, due to a lack of updates in primary school-aged children, the use of different methodologies and an absence of standard protocols, they do not allow the current situation to be assessed or changes to be monitored in Poland. The prevalence estimates derived from these studies may differ due to the use of various anthropometric methods (objective measurements or self-reported data). This problem might be aggravated by other methodological differences, for instance, year of the study, population studied (sample size, age group and sex), representativeness (local or national), varied selection of children (random or not random) and also the methods used for assessing and defining overweight and obesity (criteria, cut-off points and reference tables) (16).

To address these issues, the Childhood Obesity Surveillance Initiative (COSI) system was introduced in 2016 in Poland. The World Health Organization (WHO) European COSI programme was established in 2007 by the WHO European Region countries in response to the need to create a unique research system that would enable the systematic collection and analysis of data on the incidence of obesity among population of primary school children and to lay the foundations for the creation of an appropriate policy for the region to prevent childhood obesity (17). The initiative originally included 13 countries in the WHO European Region. In the following years, the number of countries grew systematically. Thirty-five countries of the WHO European Region participated in the most recent, fourth round of the COSI study, conducted in the school year 2015–2016. In 2016, for the first time, data collection was also organised in Poland. Currently, according to WHO information, 44 countries are involved in the COSI initiative (Table 1) (18).1According to the countries participating in the study in 2018–2019, the source was derived from the internal documents (not publicly available) of the COSI network.

**Table 1:** COSI countries from 2007 to 2019

**Country**	**Round of data collection**				
	**2007–2008**	**2009–2010**	**2011–2012**	**2015–2017**	2018–2019^***^
Albania			X^*^	X	X
Armenia					X
Austria				X	X
Belgium	X	X	X		
Bulgaria^**^	X	X	X	X	**X**
Croatia				X	X
Cyprus	X	X		X	**X**
**The Czech Republic**	X	X	X	X	X
Denmark				X	X
Estonia				X	X
Finland				X	X
France				X	
Georgia				X	X
Germany					X
Greece		X	X	X	**X**
Hungary		X		X	X
**Ireland**	X	X	X	X	X
**Italy**	X	X	X	X	X
Kazakhstan				X	
Kyrgyzstan				X	X
**Latvia**	X	X	X	X	X
**Lithuania**	X	X	X	X	X
**Malta**	X	X	X	X	X
Monaco					X
Montenegro				X	X
The Netherlands					
**Norway**	X	X	X	X	X
Poland				X	X
**Portugal**	X	X	X	X	X
Republic of Moldova			X		
Romania			X	X	X
The Russian Federation				X	X
San Marino			X	X	X
Serbia				X	X
Slovakia				X	X
**Slovenia**	X	X	X	X	X
Spain		X	X	X	X
Sweden	X	X		X	X
Tajikistan				X	X
The Former Yugoslav Republic of Macedonia		X	X	X	X
Turkey			X	X	X
Turkmenistan				X	X
Uzbekistan				X	

* Country participation in the specific round of data collection

** Bold font used for countries which participated in all rounds of data collection

*** Final list of participating countries will be confirmed after completion of the 2018–2019 COSI study round.

The study in Poland is administered under the supervision of the Institute of Mother and Child in Warsaw,2Principal Investigator of the WHO COSI Poland: Anna Fijałkowska, Associate Professor and Head, Department of Science, Institute of Mother and Child, Warsaw, Poland. Research team: Anna Oblacińska, Associate Professor; Joanna Mazur, Associate Professor; Magdalena Korzycka MA; Edyta Kolipińska MA; Maria Jodkowska PhD; Anna Dzielska PhD; and Dorota Zawadzka PhD. with the support of the WHO Country Office for Poland. The first round of the study was conducted in 2016 under the National Health Program for 2016–2020. The second round of research in Poland is currently being implemented.

## Aim

The article aims to present the main assumptions of the COSI methodology and to describe the process of standardisation used in the research procedures for data collection in Poland in the 2016 study round, as well as the implemented changes for 2018.

### COSI System – The Purpose and Objectives

The COSI is a unique system consisting of systematic data collection, analysis, interpretation and dissemination of results to monitor an important public health problem, namely, excessive body weight in children. The system involves the weight and height measurements of primary school children aged 6–9 years.

The research involves applying a standardised methodology and conducting research based on strictly defined rules in each Member State, according to the international research protocol (19). Applying these rules results in delivering the relevant data and enables both the progress of the obesity epidemic to be tracked and inter-country comparisons to be made. Although individual countries can develop systems adapted to local conditions, surveys must be collected using a standardised methodology in accordance with the agreed joint protocol to provide relevant and comparable data (19). An international protocol is developed for each survey round. The protocol contains instructions about the mandatory and voluntary requirements for research preparation and conduct, as follows: the period of data collection, sampling strategy, population age and size within the national samples, research instruments and variables, translation procedures for questionnaires, method of conducting anthropometric measurements and the tools used for this purpose, training of examiners, data entry, data management and research ethics procedures. The basic principles of the COSI system in Member States recommend creating a simple national system and integrating it with other existing systems of health care, as well as anthropometric and dietary systems.

A *mandatory objective* for each round of data collection is to measure the following:

- weight, height and body mass index (BMI);
- the prevalence of underweight, normal weight, overweight and obesity, as well as the median and mean BMI;
- changes over time in the prevalence of overweight and obesity and mean BMI in comparison to the previous cohort of children in the same age range;
- specific characteristics of school nutrition and physical activity environment.

Countries may extend the mandatory anthropometric measurements and use optional research tools and variables to measure and collect the following:

- waist circumference and hip circumference;
- concomitant diseases;
- nutritional behaviour, physical activity patterns and sedentary behaviours;
- school data, data on parents (additional form for parents);
- other national data (e.g. in Poland, blood pressure was measured in all children participating in the study).

## Study Population and Sampling Procedure

### Participants

Following the COSI protocol, measurements are carried out among primary school students. Countries can select one or more age groups from the following: 6.0–6.9, 7.0–7.9, 8.0–8.9 or 9.0–9.9. Children in Poland aged 8.0–8.9 years (the period between the eighth and ninth birthdays) were examined under the study in 2016 and 2018.

### Sampling frame in COSI Poland

It is recommended that the national sample size is close to, or more than, 2,300 children per age group, selected from a minimum of 2,800 participating pupils.

Provinces in Poland were selected according to their sizes and economic development levels. Primary schools were randomly chosen from the database of primary schools available from the website of the Ministry of National Education in the list of schools and centres by type.3Education Information System (at Education IT Centre) – http://www.cie.men.gov.pl/index.php/sio-wykaz-szkol-i-placowek/27-wykaz-wg-typow.html; accessed: 8 September 2015. Provinces were selected with the probability of selection being proportional to the population study years (2016 and 2018). Changes to the education system in Poland, in 2016, affected the heterogeneity of age groups in individual school classes (20), and hence, usually two classes in each primary school (the 2nd and 3rd grades) were selected to participate in the study. Data on total class number in each school were collected to calculate weights.

### Characteristics of final sample

A nationwide survey was carried out in November–December 2016 in 135 schools in nine provinces (37 poviats) of Poland. A group of 3,408 children from the 2nd and 3rd grades of primary school were examined. The final sample consisted of children for whom the completeness of data, in terms of the key variables, was confirmed: informed consent for participation, age, gender, height and body weight. Forms were completed by 2,298 parents (87% of the sample implementation).

The second round of study began in the final quarter of 2018 and was finished and filed at the end of the year. The survey sample was extended to 12 provinces (three provinces more than in the previous round of research). Survey was carried out in 140 schools. A group of 2691 pupils aged 8 years from the 2nd grade of primary schools were examined. Forms were completed by 2,450 parents (91% of the sample implementation).

**Figure 1:**
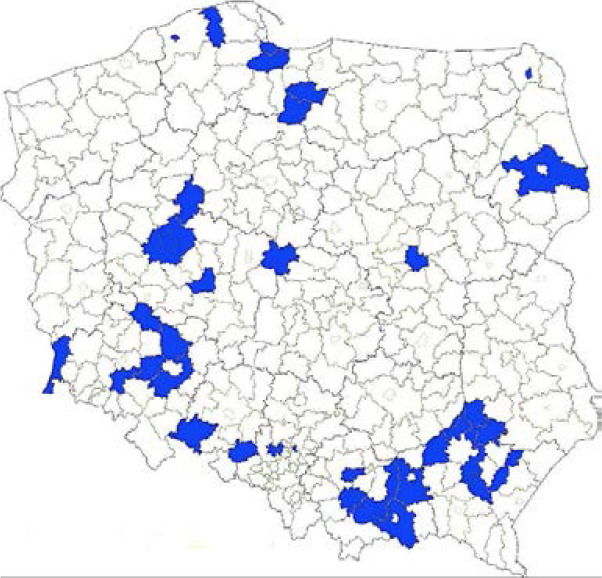
Geographical distribution of schools in Poland participating in COSI 2016.

**Figure 2:**
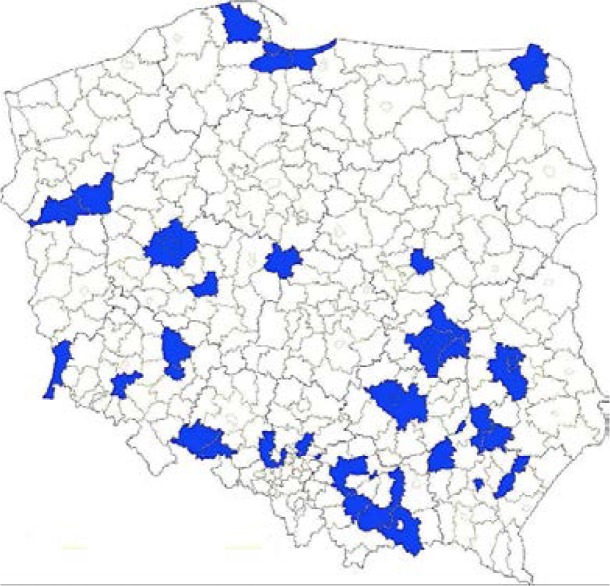
Geographical distribution of schools in Poland participating in COSI 2018.

The geographical distributions of the sample in COSI 2016 and 2018 are presented in Figures 1 and 2, respectively.

## Study Design

The COSI protocol uses a semi-longitudinal design, with repeated cross-sectional samples. Optionally, a prospective cohort design is permitted, with follow-up of the initial sample for one round. In 2016, in Poland, the research was conducted according to the basic recommendations.

### Study Instruments

The COSI instruments comprise three types of questionnaires (forms) that are used to create the national survey:

- mandatory instruments: (1) Child Record Form, completed by the examiner; and (2) School Record Form, completed by the school nurse or school director;
- voluntary instrument: (1) Family Record Form, completed by the parents or guardians.

Variables used in the Polish COSI survey in 2016 included mandatory anthropometric measurements (weight, height and waist and hip circumferences), as well as data from measurements of diastolic and systolic blood pressure as national variables. The optional questionnaire for parents was also used, which included questions about, among other things, diseases occurring in the family, family lifestyle, as well as the child’s physical activity and eating behaviour. The particular thematic areas included in the forms are described in Table 2.

**Table 2:** Research areas and items of the COSI study in Poland in 2015–2016 and 2017–2018 survey round

**Research area**	**Main indicators**	**Survey round^1^**	
		**2015–2016**	**2017–2018**
Child Record Form (MF)**2**			

Child identification data	Date of birth	M	M
	Age, in months	V	V
	Sex	M	M
	Place of residence	M	M

Anthropometric examination procedure	Date of measurement	M	M
	Time of measurement	V	V
	Breakfast consumption on the day of measurement	M	M^*^
	Meal before measurement	M	M
	Child consent	M	M
	Reason for child refusal	V	V
	Type of clothes during measurement	M	M

Measurement items	Body weight (kg)	M	M
	Body height (cm)	M	M
	Waist circumference (cm)	V	V
	Hip circumference (cm)	V	V
	Blood pressure (mmHg), percentiles	N	N
	Heart rate (bmp)	–	N

Parent Record Form (VF)**2**			

General information about child	Sex	M	M
	Date of birth	M	M
	Birth weight (g)	M	M
	Whether breastfed	M	M^*^
	Duration of exclusive breastfeeding	M	M^*^
	Child's order (e.g. first, second, only child, etc.)	–	N
	Pregnancy order	–	N
	Length of pregnancy	M	M^*^

Child's behaviour characteristics	Distance from school to home	M	M
	Transport from/to school (e.g. by bus, car, foot, etc.)	M	M^*^
	Participation in sport clubs and extracurricular physical activities, hours per week	M	M
	Time spent on physical activity	M	M
	Time spent on homework	M	M
	Sedentary behaviours (screen time)	M	M^*^
	Breakfast consumption	M	M
	Food frequency questionnaire	M	M^*^
	Sleep duration (school days)	M	M
	Place of hot meal consumption	N	N
	Type of diet (e.g. vegetarian, lactose-free)	N	N

Family environment	Joint family-based sport activities (mother, father and siblings)	–	N
	Other joint family activities	–	N
	Family meals (breakfast, dinner and supper)	–	N
	Parental feeding practices (e.g. pressure to eat, restriction and monitoring of education)	–	N

	Family communication	–	N

Parent/family members’ health	Diagnosed diseases (high blood pressure, hormonal problems, diabetes and altered cholesterol levels)		
	Parents’ weight and height	M	M
	Self-rated health	–	N
	Life satisfaction	–	N
	Vitality	–	N
	Spirituality	–	N
	Purpose in life	–	N

Parent/family members’ health and risk behaviour	Tobacco smoking (lifetime/past 12 months)	–	N
	Alcohol use	–	N
	Physical activity	–	N

Child health	Chronic conditions and medicine use	–	N
	Child's weight perception	–	N

Family's socioeconomic status	Perceived family wealth	M	M
	Family structure	–	M
	Number of family members younger and older than 18 years of age	M	–
	Parents’ education	M	M
	Parents’ work status	M	M^*^
	House/flat ownership	M	–

Other information	Child's/parent's country of birth	–	M
	Parents’ language of communication	–	M

School Record Form (VF)**2**			

Identification data	Respondent position	M	M
	School name	M	M
	Address	M	M
	School location	M	M

Information on participating classes/pupils	Number of classes selected per grade	M	M
	Number of pupils selected/examined	M	M
	Number of pupils selected but not examined (reason of refusal)	M	M

School environment	Physical education lessons	M	M
	School playground/gym	M	M
	Open access to school physical activity infrastructure	V	V
	Extracurricular sport activities/classes at school	V	V
	Access to school bus	V	V
	Road safety around school	V	V
	Food and beverages availability	M	M
	Health promotion/health education at school	M	M
	School canteen	M	M
	School shop/buffet	V	V
	Vending machine	M	M

	Prohibition of junk food advertisement at school	M	M

1 Question type was marked with a letter according to the following key: M: mandatory; V: voluntary; N: national.

2 According to the COSI protocol, accessible forms should be used as mandatory or voluntary. The type of form was marked with a letter according to the following key: MF: mandatory form; VF: voluntary form.

* The question was modified by the COSI Coordination Centre in the 2017–2018 protocol.

### Translation Procedure

The tool translation process in the COSI programme followed the procedure specified by the WHO. According to these standards, all forms and research documentation used in the Polish COSI study (measurement protocol, school form and instructions, the form with the letter and the consent form for the parents and letters to the schools) were translated into Polish, following the indicated recommendations. Standard procedure included the following: forward translation, expert panel back-translation, pretesting and preparation of the final version of all documents in Polish.

### Ethics

Consent for the study was obtained from the Bioethics Commission at the Institute of Mother and Child in 20154Opinion of the Bioethical Commission No. 22/2015 (22.11.2015). and 2018.5Opinion of the Bioethical Commission No. 51/2018 (14.11.2018). In both cases, the opinion of the Commission was obtained for the research procedure, research tools and the method of obtaining consent from parents, together with the consent form. The active, written consent of the parent and the child was required for admission to the study.

## Study Procedure

### Examiners and their duties

Implementation of the COSI survey was carried out in cooperation with provincial coordinators and school nurses. The invited coordinators were team managers providing nursing care for students in schools.

A 2-day training programme was held in November 2016 for the provincial coordinators. The training was carried out by a Polish team from the Institute of Mother and Child and by representatives from the WHO office, with the participation of foreign experts. The thematic scope of the training covered, among other things, the main assumptions of the project and the methodology for anthropometric measurements, research tools and the tasks of the provincial coordinators and school nurses. Provincial coordinators supervised the correct implementation of COSI surveys in schools in their region. They were in constant contact with the examiners (school nurses) and helped them to solve emerging problems, answered questions, and in cases of doubt, contacted project coordinators at the Institute of Mother and Child.

The examiners were responsible for the following:

- measurement of children’s anthropometric parameters (weight, height, as well as waist and hip circumferences) and also systolic and diastolic blood pressure;
- filling out the child measurement form;
- delivering the school form to the school principal or complete it;
- delivering the appropriate forms to parents (consent form and parent form);
- transfer of documentation and completed forms to the project coordinator at the Institute for Mother and Child.

### Measurement procedures

Measurements were conducted by trained school nurses according to the COSI protocol procedures using standardised measurements. SECA 878 – mobile electronic scales were used, with coupled mobile stadiometer SECA 217 (Seca GmbH & Co.), which are recommended and meet the requirements of COSI procedures. Other type of measurement equipment was not specified in protocol. Centimetre tape and electronic sphygmomanometers using cuffs in sizes suitable for children aged 8 years (OMRON, different types, not specified), both from the schools nurse’s office, were used. Each measurement was performed twice, and the final value was the arithmetic mean of the two measurements. The result calculated in this way was entered by the nurse into the child’s measurement form.

- Body height was measured to the nearest 1 mm (0.1 cm), without shoes, in Frankfurt horizontal plane.
- Body weight was determined to the nearest 100 g (0.1 kg), with the children in minimal clothing (wearing only underwear) and no shoes.
- Waist and hip circumferences were measured to accuracy of 1 mm (0.1 cm).
- Blood pressure (systolic and diastolic, mmHg) measurements were made according to the recommendations of the screening test for the detection of elevated arterial pressure (21). Children were examined in a sitting position after a few minutes of rest. The measurement was carried out twice, and the arithmetic mean of the two measurements was recorded as the final result.

The data from the received questionnaires were coded by a team from the Institute of Mother and Child, using the Open Clinica system (2016) or EpiData forms (2018). The 2016 database was prepared based on the WHO COSI Guidelines on Data Processing and Cleaning. The database was subjected to the verification procedure by the WHO Regional Office for Europe. The same procedure was implemented for the data 2019.

### Classification of key variables

Following the COSI protocol, the criteria developed by WHO were applied to define overweight and obesity using key variables (weight and height). Cut-off points were used to calculate the BMI *z*-score appropriate for sex and age (BMI-for-age), in accordance with WHO 2007 recommendations (22), and the ‘WHO Anthro’ macro for SPSS was used for the calculations (23). BMI was calculated as a measure of the relative weight index. Overweight was defined as the percentage of children with BMI +1 standard deviation (SD) *z*-score, and obesity was defined as children having *z*-score above +2 SD. Such cut-off points corresponded to values of BMI = 17.7 kg/m^2^ for boys and BMI = 18.0 kg/m^2^ for girls in cases of overweight and BMI = 20.1 and 21.0 kg/m^2^, respectively, in the case of obesity. The assessment was made in semi-annual age ranges.

## Summary

The implementation of the COSI system for the assessment of the occurrence of overweight and obesity in early childhood in Poland is an example of a well-designed surveillance system based on high-quality standards. Experience from the first edition of the Polish COSI study (2016) allowed to modify the data collection system, the manner of conducting trainings and the method of recording measurements by nurses. Data obtained from cyclical measurements carried out with the same method and according to a unified protocol in several dozen European countries will enable a reliable assessment of the scale of the phenomenon, its changes over time and an assessment of differences between and within countries in the European Region. In addition, examining the prevalence of overweightness and obesity among children can contribute to the design of more-effective interventions targeting this age group.
